# Fermentation Efficiency of Genetically Modified Yeasts in Grapes Must

**DOI:** 10.3390/foods11030413

**Published:** 2022-01-31

**Authors:** Konstantina Kassoumi, Penny Kousoulou, Dimitrios Sevastos, Sotirios-Spyridon Vamvakas, Konstantinos Papadimitriou, John Kapolos, Athanasia Koliadima

**Affiliations:** 1 Department of Chemistry, University of Patras, 26504 Patras, Greece; kkasoumi14@gmail.com (K.K.); penny.kousoulou96@gmail.com (P.K.); dsevastos@gmail.com (D.S.); 2 Department of Nutritional Science and Dietetics, University of the Peloponnese, 24100 Kalamata, Greece; svamvakas@uop.gr; 3 Department of Food Science and Technology, University of the Peloponnese, 24100 Kalamata, Greece; k.papadimitriou@uop.gr (K.P.); i.kapolos@uop.gr (J.K.)

**Keywords:** alcoholic fermentation kinetics, genetic modification, *Saccharomyces cerevisiae* cell production, life cycle of yeast, currents, yeast growth phases, reversed-flow gas chromatography, separation techniques

## Abstract

Winemaking is a stressful procedure for yeast cells. The presence of high levels of carbohydrates at the beginning of the fermentation and the subsequent increase of ethanol levels alongside with other environmental factors force the cell to undergo a continuous adaptation process. Ideally, yeast strains should be able to adapt to this changing environment fast and they must be able to ferment at low temperatures with the highest possible fermentation rates. Additionally, the balanced utilization of glucose and fructose—the two major hexoses in grapes—is also important as any residual fructose may confers unwanted sweetness. As proteins, Msn2/4 are known to play pivotal roles in cell stress response, the question that arise regards the differentially cell response driven by specific point mutations in these two proteins, and the subsequent effects on alcoholic fermentation. Four different mutants in which serine residues have been replaced by alanine are studied in this paper. Our results indicate that substitution at position 533 of Msn4 protein (W_M4_533) significantly increases the fermentation rate even at low temperatures (12 °C), by lowering the fermentation’s activation energy. Similar results but to a lesser extent were obtained by the S582A substitution in Msn2 protein. In addition, W_M4_533 seems to have a more balanced utilization of must hexoses. From the present work it is concluded that genetic modification Msn2/4 represents a promising procedure for shortening the fermentation time, even at low temperatures, which in many cases constitutes an important technological requirement.

## 1. Introduction

Indigenous yeasts are responsible for the fermentation process during traditional winemaking. In order to achieve higher fermentation rates, selected yeast strains are used. The inoculation of grape juice with selected starter cultures of *Saccharomyces cerevisiae* resulting in rapid and consistent fermentation, is a concept which has become widely accepted [1,2].

With the progress of biotechnology and molecular biology, many changes were brought in traditional wine making procedures. Specific *S. cerevisiae* strains can be generated carrying desired alteration in one or more proteins resulting in the improvement of alcoholic fermentation characteristics. Msn2/4 proteins are transcription factors regulating stress-response of yeast cells [3]. Their activation is control by several kinases [4]. Protein kinase A (PKA) which is a heterotrimer of tpk1/2/3, is a kinase which phosphorylates several proteins. These phosphorylation events result in the induction or suppression of the target proteins. Msn2/4 are targets of PKA and their phosphorylation at specific serine residues results in their inability to translocate to the nucleus [5]. The presence of glucose in the fermented medium activates PKA anchoring Msn2/4 to the cytoplasm [6]. The complete depletion of PKA and the subsequent growth arrest results from a maladjusted metabolic state triggered by Msn2-dependent gene expression changes [7].

During must fermentation, sugars and ethanol are both present, with the concentration of the later one continuously increasing against the concentration of the sugars. There is a concentration threshold for both of these, where the inactivation of Msn2/4 is reverted. It has been shown that yeast cells lacking Msn2 and Msn4 exhibit prevalent repression of glycolytic genes and a significant delay of acetyl-CoA accumulation and reentry into growth from quiescence as well as impaired expression of genes involved in the fatty acid oxidation and present an increment in triacylglycerol and steryl ester levels [8,9]. As the depletion of these transcription factors seems to affect the metabolic function of the cell, a more targeted intervention to PKA/Msn2/4 system may contribute to the improvement of alcoholic fermentation. In this concept, there have been a couple of studies from our lab, in order to partially inhibit the Msn2/4 inactivation through the PKA phosphorylation and subsequently help yeast cells to ferment more efficiently and faster, using synthetic substrate. In these studies, several Msn2/4 serine residues that had been thought to undergo phosphorylation and hold these transcription factors to the cytosol, have been replaced by alanine. Some of these replacements were shown to be beneficial in ethanol production [10,11].

In this study, we tried to validate these results using as substrate must from reconstituted Corinthian currants, which are used for the production of ethanol at an industrial scale as well as in wine making. In addition to this, we also wanted to test a new modification on Msn4. Finally, an effort was made to calculate the physicochemical characteristics of the fermentation’s efficiency of each strain.

## 2. Materials and Methods

### 2.1. Materials

All chemicals used were of reagent grade. DNA HiFi Polymerase was from KAPA Biosystems (Wilmington, MA, USA)). Restriction enzyme (DpnI) were from New England Biolabs Inc. (Rowley, MA, USA)).

### 2.2. Yeast Strains, Media, and Growth Conditions

All strains are derivatives of W303-1a and are shown in Table 1. Standard yeast culture methods were used. Yeast cells were grown in YPD (1% *w*/*v* yeast extract, 2% *w*/*v* bactopeptone, 2% *w*/*v* glucose) or synthetic medium lacking uracil or supplemented with 5-fluoroorotic acid (5-FOA) at 30 °C [12].

### 2.3. W_M4_533 Mutant Generation

The generation of a yeast strain carrying the modification S533A on Msn4 was performed as described previously [10]. Briefly, cloned MSN4 gene in pUC19 plasmid was used for the genetic modification using the polymerase chain reaction (PCR) site-directed mutagenesis approach. The primers MSN2_533_F and MSN2_533_R as shown in Table 2 were used. PCR product was treated with Dpn-I restriction endonuclease for the complete removal of the template DNA [13] The Dpn-I digested DNA used for the transformation of TOP-10 chemically competent bacterial cells.

Wild-type MSN4 gene was replaced by a functional PCR generated URA3 gene in W303-1a genome resulting in the creation of W303-1a_ΔMSN4 yeast strain. Transformed cells with successful gene replacement were selected by plating in minimal medium lacking uracil. The strain W_M4_533 was constructed by the replacement of the previously inserted functional URA3 gene in W303-1a_ΔMSN4 strain with MSN4_533 gene, and the selection of the transformed cells was achieved by plating them on a minimal medium supplemented with 5-FOA at concentration of 1 mg/mL. All required transformations were carried out using the lithium acetate method as described by Ito and co-workers [14]. PCR was used for verifying the removal of MSN4 wild-type gene as well as for the incorporation of the mutated form using as primers MSN4-URA3_F/MSN4_URA3_R and MSN4_F/MSN4_R, respectively.

### 2.4. Must Preparation, Inoculation and Sampling

Commercially available Corinthian currants were used as a raw material. To 1 L of tap water at approximately 40 °C, 1 kg of currents was added. The currants were macerated for about 20 min in order to extract the currant broth. During the process the sugar content was monitored via Baume determination. The must used for the alcoholic fermentation analysis was of 13.4 °Be density [15].

Two hundred mL of must in 250 mL Erlenmeyer flasks were inoculated with 2 × 10^8^ CFU from freshly prepared yeast cultures and incubated at 12, 18 & 23 °C, in an IPP55 incubator (Memmert GmbH + Co. KG, Schwabach, Germany), until the end of the fermentation at the appropriate temperature. Samples were collected during the fermentation, every 0.5–1 °Be decrease of the fermented substrate. The end of fermentation was determined by the zeroing of the °Be. The temperature of 23 °C was selected for near optimum yeast growth and function, while the temperature of 18 °C as a common temperature for the wine fermentation [15]. One of the major concerns of wine makers is to be able to ferment under temperatures as low as possible in order wine to maintain as much as possible the grapes’ aromatic characteristics. Bearing this in mind, we chose to test the ability of the strains to ferment at 12 °C.

### 2.5. Reverse Flow Gas Chromatography (RFGC)

A model 8A gas chromatograph (Shimadzu, Kyoto, Japan) equipped with a flame ionization detector (FID) was used for the separation and quantitation of ethanol. The system and the procedure have been described thoroughly elsewhere [16] and it is described here only briefly.

A sampling column with the two sections of lengths *l* = *l*’ = 100 cm and the diffusion column with length *L*_1_ = 43 cm were used. At the lower end of the diffusion column, a glass vessel (*L*_2_ = 5 cm) containing the fermentation mixture was connected. An analytical column (2 m × 1/4 in × 2 mm glass) packed with 5% Carbowax-20 M and 80/120 mesh Carbopak BAW, which was kept at 115 °C, was placed before the detector in order to separate ethanol from alcoholic fermentation’s by-products. The temperature of the detector was set at 150 °C whereas the temperature of the chromatographic cell was 75 °C. The experiments were conducted under constant flow rate (20 mL min^−1^) using helium as carrier gas.

An aliquot of 0.5 mL of the fermentation mixture was added in the glass vessel at constant temperature and pressure. By the time a concentration–time curve appears, the chromatographic procedure begins by reversing the carrier gas flow direction, through a Shimadzu valve, for 6 s. This period of time is shorter than the gas hold-up time in both sections *l* and *l*’. The height *h* of each sample peak is proportional to the concentration *c*(*l’*, *t*) of the solute in the sampling column at *x* = *l*’ and at time *t*, when the reversal occurs [17]. The above procedure was repeated in different fermentation periods (*t*_x_) from the initial to the final phase of the fermentation process and the corresponding values of *H*_x_ were calculated. After a mathematical model we conclude to the following equation [18]:

ln(*H*^0^_x_ − *H*_x_) − ln *H*^0^_x_ − *kt*_x_ (1)

where *H*^0^_x_ is the value of the height of the sampling peak, *H_x_*, at the end of the fermentation and *t*_x_ the fermentation time. By plotting ln(*H*^0^_x_ − *H*_x_) against *t*_x_ the values of the rate constants for the fermentation process (*k*), can be calculated. Finally, solutions with different ethanol concentration (%, *v*/*v*) in triply distilled water were placed into the glass vessel at the end of the diffusion column and the values of *H*_x_ were measured. These values and their corresponding ethanol concentrations were plotted and a calibration curve (slope = 1.202 cm M^−1^, intercept = 0.041 cm and R^2^ = 0.999) was obtained. From the calibration curve and by measuring the height of the sample peaks during the fermentation process we can find the unknown ethanol concentrations of each fermentation mixture.

### 2.6. Activation Energy Determination

Graphic procedures based on the Arrhenius model have been used in a lot of studies in order to calculate thermodynamic and kinetic quantities of enzymatic and cellular processes [19]. A physicochemical analysis based on Arrhenius model was conducted. Calculations were carried out for the determination of the activation energy of the fermentation processes for each yeast strain. The activation energies were calculated from the following equation which is based on the Arrhenius equation [20]:

(2) $$\ln\left(k\right)=\ln\left(A\right)-\frac{E_{a}}{R}\;\frac{1}{T}\;$$

where *k* is the reaction rate constant, *A* the pre-exponential factor, *E*_a_ the activation energy of the reaction, *R* the universal gas constant in J K^−1^ mol^−1^, *T* the absolute temperature in kelvin (K). Plotting of the ln(*k*)· *R* vs. 1/*T* the resulting slope of the linear regression for each strain is equals to the activation energy of each fermentation phase for each strain.

### 2.7. HPLC Analysis

The glucose concentrations were determined using an LC-20A Prominence HPLC system (Shimadzu) equipped with a refractive index detector (RID-20A/20Axs), a system controller (CBM-20A/20Alite), a column oven (CTO-20A/20 AC), an autosampler (SIL-20AHT/20ACHT) and an on-line degasser (DGU-20A3/20A5). Water-acetonitrile (20:80) at a flow rate of 3 mL/min under isocratic conditions was used as mobile phase and the total run time was 8 min. Separation was accomplished on a Luna 5μ NH_2_ 100 Å column (Pickering Laboratories Inc., Mountain View, CA, USA). The column temperature was set at 40 °C and a volume of 10 μL was injected for each sample in triplicate. Standard solutions of 0, 20, 50, 100, 200, and 300 (g L^−1^) of glucose were prepared and used for the construction of the standard curve used for the determination of the sugar concentration in fermentation samples.

Glucose’s and fructose’s calculated concentrations of samples during fermentation, were used for the determination of ln(*C*_sugar,x_). By plotting ln(*C*_sugar,x_) against *t*_x_, the values of the rate constants for the consumption of process (*k*), can be calculated by the slope of the resulting line for every phase of the alcoholic fermentation when linear convergence is applied. The total hexose content was calculated by adding the concentration of glucose and fructose. The ln(_hexose,x_) plotted against *t*_x_, and the respective slopes were calculated as stated above.

### 2.8. Statistical Analysis

In order to test if the observed differences on calculated slopes are significant, the procedure described below was followed:

- The standard error of calculated slope was determined.
- *t*-value was calculated
- The students t-distribution (*p*) was used to test the hypothesis of equal slopes.
- Statistical analyses were performed using Microcal Origin v.9.0 (Microcal Software Inc., Northampton, MA, USA).

## 3. Results

### 3.1. Generation of W_M4_533

**Figure 1:**
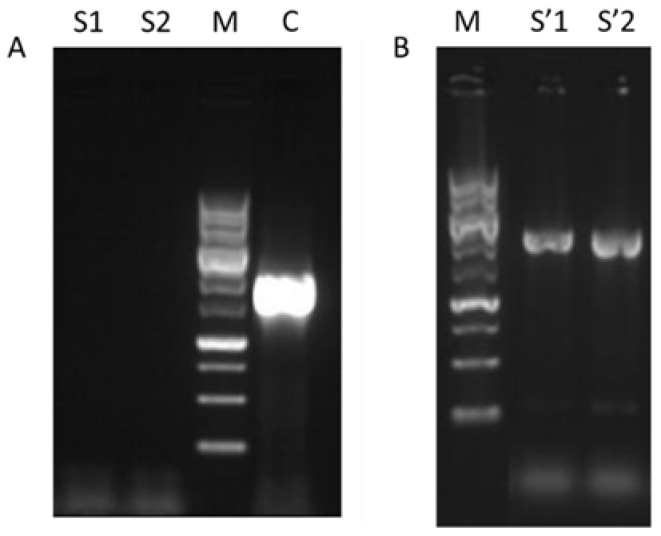
Electrophoresis of PCR products for W_M4_533 validation. (**A**) Validation of MSN4 deletion (S1, S2: W_M4_URA3 candidate colonies 1 and 2 respectively, M: Molecular weight marker (MWD-1, Nippon Genetics), C: W303-1a). (**B**) Validation of MSN4_S533A gene insertion for W_M4_533 generation (M: Molecular weight marker, S’1 and S’2: W_M4_533 candidate colonies 1 and 2 respectively).

The insertion of mutated MSN4 gene at position 533 in yeast cells was performed in two steps. In the first step, the wild type MSN4 gene was substituted with functional URA3 gene (W_M4_URA3). The confirmation of the substitution was performed with PCR using as primers MSN4_F and MSN4_R. The positive result of this PCR was the non-formation of a product. Alongside with the genomic DNA isolated from candidate transformed strains, W303-1a strain’s genomic DNA was also used, and the expected product of the later was a single band of ~1900 bp (Figure 1A). In the second step the insertion of the mutated copy of MSN4 substituted the previously inserted URA3 gene (W_M4_533 strain) and the confirmation was achieved by PCR (Figure 1B)

Alcoholic fermentation is a process which stresses the cell. Once the yeast has been added to the fermentation medium, it is exposed to a stressful environment due to the high sugar concentration. The gradual production of ethanol is also a stressful condition. The cell responds to this stress by activating several transcription factors, which in turn, regulate the expression of several downstream genes. Some of them are implicated in the fermentation process.

### 3.2. Determination of Ethanol Production Using RFGC

In the present work the RFGC technique was used for the kinetic study of ethanol production and the distinction of the growth phases of wild type and mutant yeast strains during the alcoholic fermentation, conducted using as substrate must derived from reconstituted dried currants.

**Figure 2:**
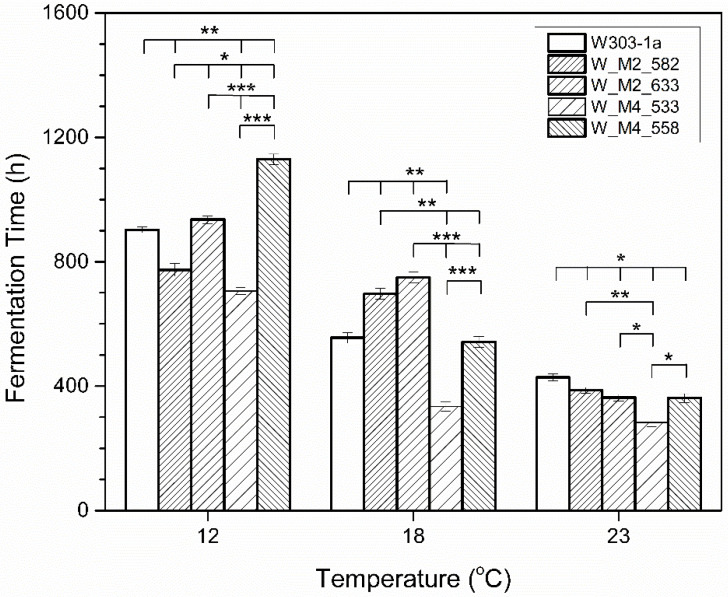
Total fermentation time (h) at several temperatures. Statistically significant differences were designated with asterisks (* *p* < 0.05, ** *p* < 0.01, *** *p* < 0.001).

Strain W_M4_533 was shown to be the quickest in completing the fermentation as shown in Figure 2. The same result was observed at all tested temperatures. Strain W-M2_582 was shown to ferment quicker than the wild type (W303-1a) at the lowest tested temperature (12 °C). Substitution of serine residue at position 558 in Msn4 as well as at position 633 in Msn2 transcription factors, resulted in a small delay in fermentation completion in all tested temperatures.

### 3.3. Kinetic Analysis

The observed differences in fermentation completion as well as in fermentation efficacy, led us to perform the kinetic analysis of fermentation as described in Section 2.5.

**Figure 3:**
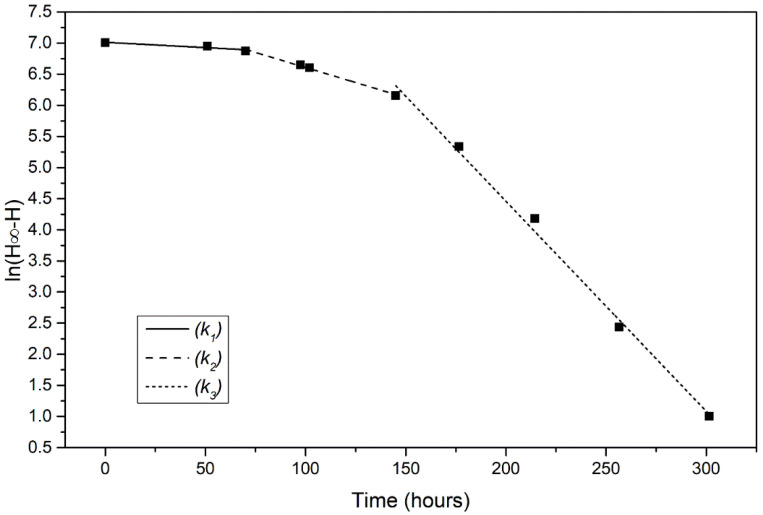
Representative plot of ln(*H*^∞^–*H*) vs. time for fermentation at 18 °C by wild type strain W303-1a. The slope of each of the three lines represents the reaction rate constants *k*_1_, *k*_2_ and *k*_3_ of the three fermentation phases.

The analysis revealed that there were three phases. The initial lag phase where the yeast is adjusting to its new environment with a minor ethanol production, the log phase during which the yeast cells begin to reproduce rapidly, the growth rate is maximum and an increase in ethanol production is shown. Finally, the stationary phase where the ethanol production is maximal. The reaction constant of each phase for every yeast strain was calculated as described in Section 2.5. Representative plots of a fermentation process conducted at 18 °C are shown in Figure 3. Each phase corresponds to a first order reaction, with the reaction rate constants for lag phase, log phase and stationery/death phase are *k*_1_, *k*_2_, and *k*_3_, respectively.

**Figure 4:**
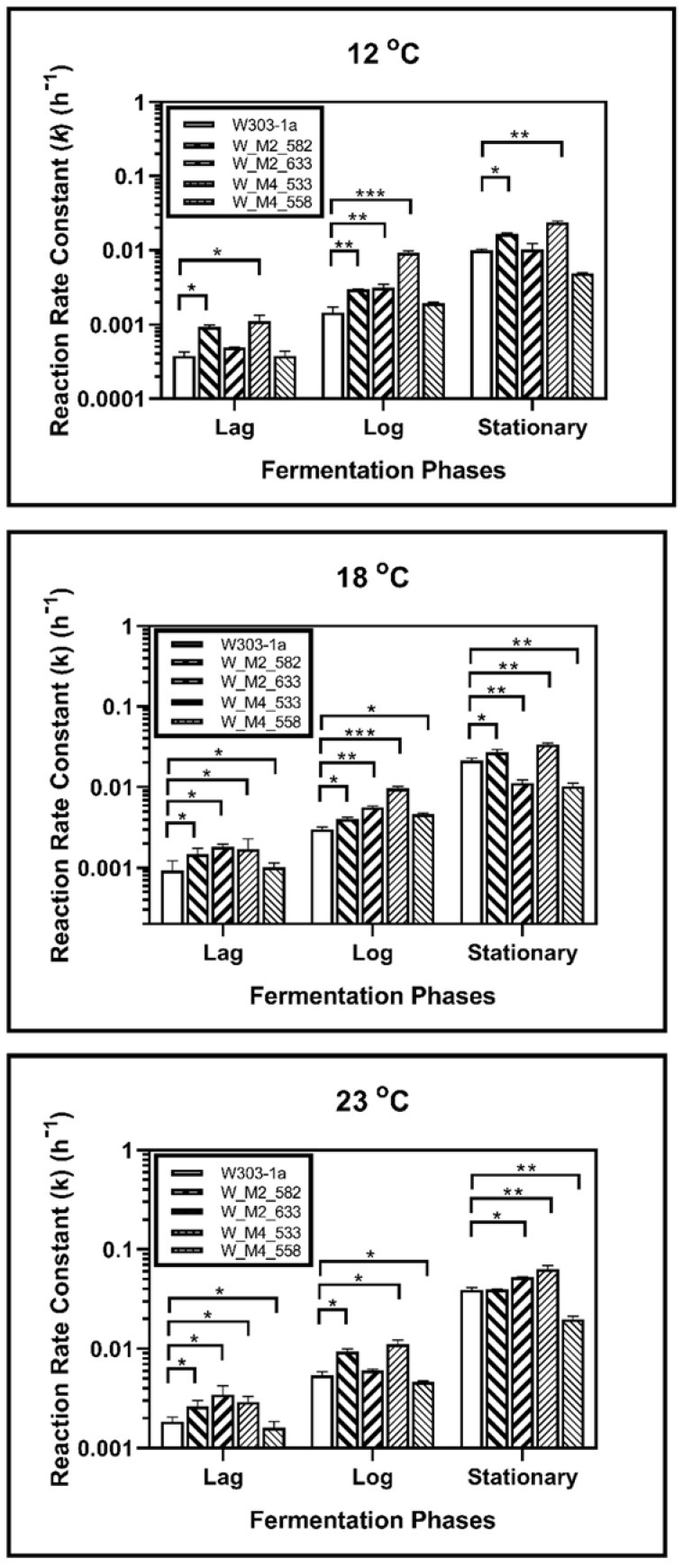
Reaction rate constants of alcohol formation during alcoholic fermentation regarding phases for each strain at 12, 18 and 23 °C. The statistically significant differences are nominated by asterisks: (* *p* < 0.05, ** *p* < 0.001, *** *p* < 0.0001).

During fermentation at 23 °C, it was revealed that the S633A substitution in Msn2 helps yeast to adapt quicker to the fermentation environment, as it showed a higher reaction rate constant during lag phase (*k*_1_). This was not observed during log phase as there was not statistically significant difference between calculated reaction rate constants (*k*_2_). During the stationary phase this strain showed a statistically significant increase in reaction rate constant (*k*_3_) suggesting a possible resistance in ethanol. The W_M4_558 strain showed a statistically significant reduction of the reaction rate constant in each phase, indicating that the serine residue in the specific position of Msn4 is probably required for higher fermentation rates. Both W_M2_582 and W_M4_533 showed statistically significant increased ability to ferment during lag and log phase, but during stationary phase only W_M4_533 maintained this feature (Figure 4).

During log phase both of the MSN2 mutants showed a better ability to ferment than wild type strain. Same results were recorded during log phase, but during stationary phase the results were different. Strain W_M2_582 showed also an increase in reaction rate constant but the ethanol production of W_M2_633 strain was significantly reduced (Figure 4).

In stationary phase, W_M4_533 showed a higher fermentation rate. W_M2_633 showed also increased fermentation rate compared to W303-1a, but significantly lower than W_M4_533. The fermentation rate of W_M2_582 strain was not changed compared to the wild type’s, and W_M4_558 strain’s one was significantly lower than the wild type’s.

Fermentation kinetic analysis at 18 °C revealed that all genetically modified strains except W_M4_558 showed a statistically significant increment in reaction rate constant compared to the wild type during the lag phase, whereas W_M4_558 showed a slightly decreased *k* value. All mutants showed k values that were increased compared to the wild type’s during log phase. A greater increment was observed for W_M4_533. The entrance of the cells into stationary phase resulted in the dominance of W_M4_533 with the W_M2_582 following. Strains W_M2_633 and W_M4_558 were shown to lag behind the wild type strain (Figure 4).

Of great interest were the results for fermentation at 12 °C. It was shown that during all fermentation stages, the substitution of serine residue with alanine at position 533, in Msn4 transcription factor, confers significantly increased fermentation rates. Strain W_M2_582 showed increased fermentation ability compared to the wild type’s, but to a significant lesser extent than W_M4_533 (Figure 4). These results indicate that the substitution S533A in Msn4 confers increased fermentation ability transforming it into a strain better adapted to fermentation at lower temperatures.

### 3.4. Activation Energy

**Figure 5:**
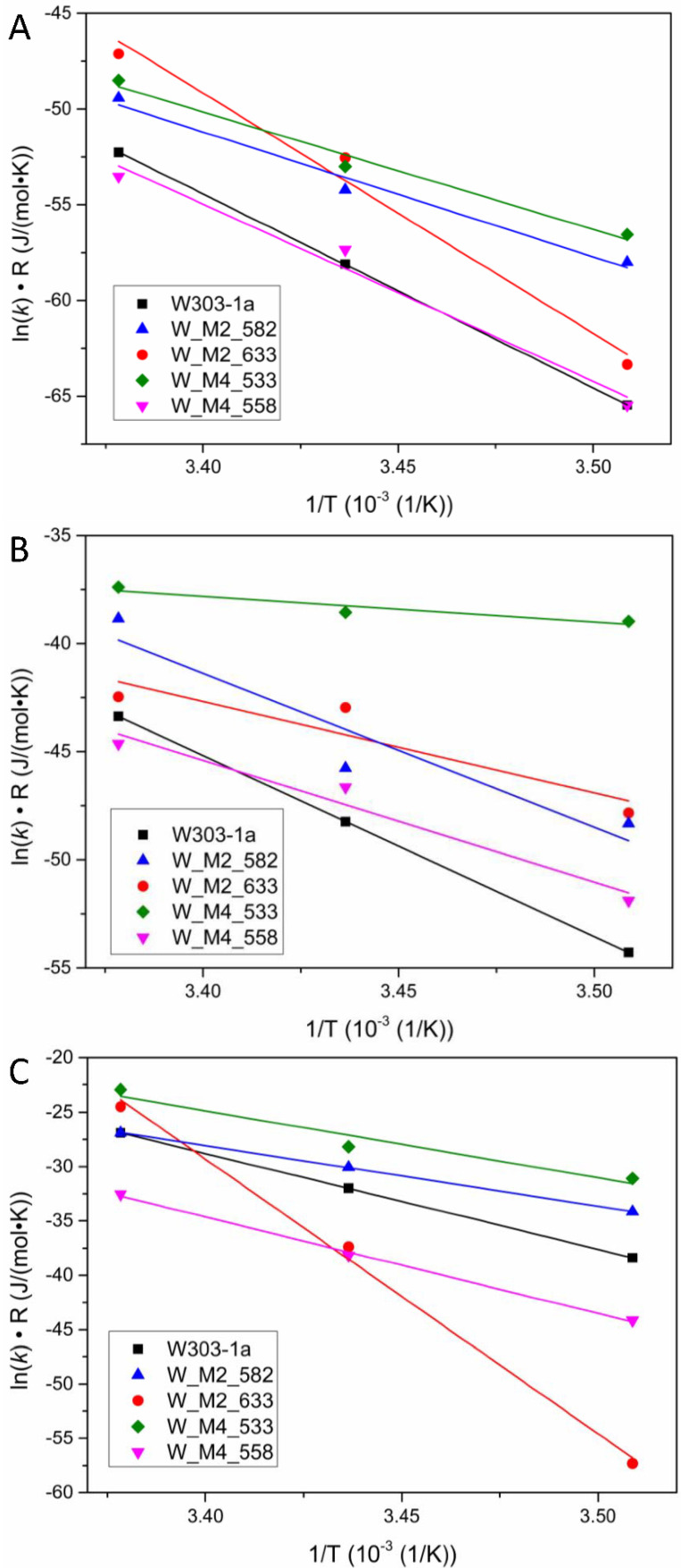
Plots of the ln(*k*)· *R* product against 1/*T*, where *T* is the temperature in Kelvin. The slopes of the resulted lines correspond to the activation energy of the reaction during each phase of alcoholic fermentation according to Arrhenius model for each studied strain. (**A**) Lag phase, (**B**) Log phase, (**C**) Stationary/Death phase.

Following the calculation methodology described in the Materials and Methods section, the plots, from which the activation energies for each strain can be calculated, are presented in Figure 5.

**Figure 6:**
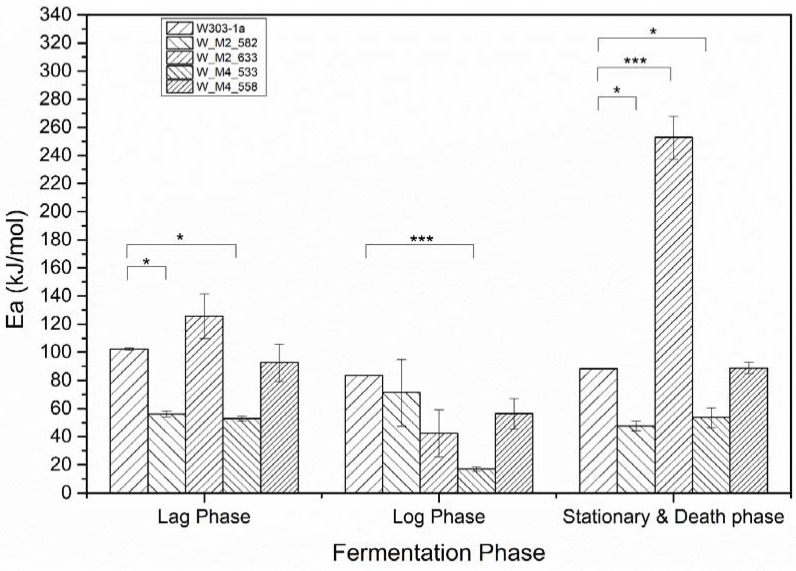
Calculated activation energies according to Arrhenius model (*: *p* < 0.05, ***: *p* < 0.001).

The resulting activation energies are presented in Figure 6. The statistical analysis by comparing the fitting lines for each strain carrying a mutation with the wild type strain (W303-1a) using the F-test, revealed that the observed differences in activation energy of the lag phase are statistically significant only for the W_M2_582 and W_M4_533. These strains showed an approximately 50% decrease in activation energy. This result clearly shows that the strains carrying the designated genetic modifications have the ability to adjusting to the new environment more quickly and efficiently than the others and show a psychrophilic character, especially the W_M4_533 strain. Regarding log phase activation energy, for once again the W_M2_582 and W_M4_533 strains were shown statistically significant decrease in calculated activation energy compared to wild type strain. The exciting result is that W_M4_533 reduces the activation energy by more than 65%, where the W_M2_582 only by 21%. This result clearly reveals that the substitution of serine residue at position 533 of Msn4 probably plays a critical role in cell function promoting its ability to grow and simultaneously produce ethanol at high rates.

At stationary phase where the cells are not multiplying but continuing the ethanol production and they are already in a stressful environment as the ethanol concentration is arising, all strains except 558 were found to have statistically different values of activation energy. Specifically, 533, 582 activation energy were lower than the wild type’s by almost 50%. The interesting result is that the 633 strain were shown a dramatically increment of activation energy. It found to be 2.5 times greater of the wild type strain. This result indicates the substitution S633A in Msn2 protein resulting in a defect in producing ethanol.

Summarizing, the overall activation energy of W_M4_533, and W_M2_582 seems to be lower than that of the wild type strain. This indicates the existence of the specific serine residues resulting in the delaying of the ethanol formation by the cells. The substitution of the serine residue at position 533 in Msn4 transcription factor confers to the yeast not only faster fermentation rates, but also the ability to adapt faster and more efficiently at lower temperatures.

### 3.5. Sugar Consumption

The major carbon source for yeast cell are carbohydrates. These are used by the cells mostly for the production of ethanol under anaerobic conditions. In a living organism all biochemical pathways are under rigorous control, in order to give the organism the opportunity to overcome any adverse condition. In addition, all fermenting sugars are involved in controlling the PKA pathway which in turn is involved in Msn2/4 translocation to the nucleus under stress condition [21]. Keeping this in mind, several questions arise, regarding the sugar consumption. Is it possible, by the presence of a different mutation on either Msn2 or Msn4, to modify the sugar consumption rate, in a way different than that of ethanol production? Do all the mutants use the available sugars in the same way? Was the reduction of the ethanol formation rate constant in W_M2_633 and W_M4_558 a consequence of a reduced ability to catabolize them?

**Figure 7:**
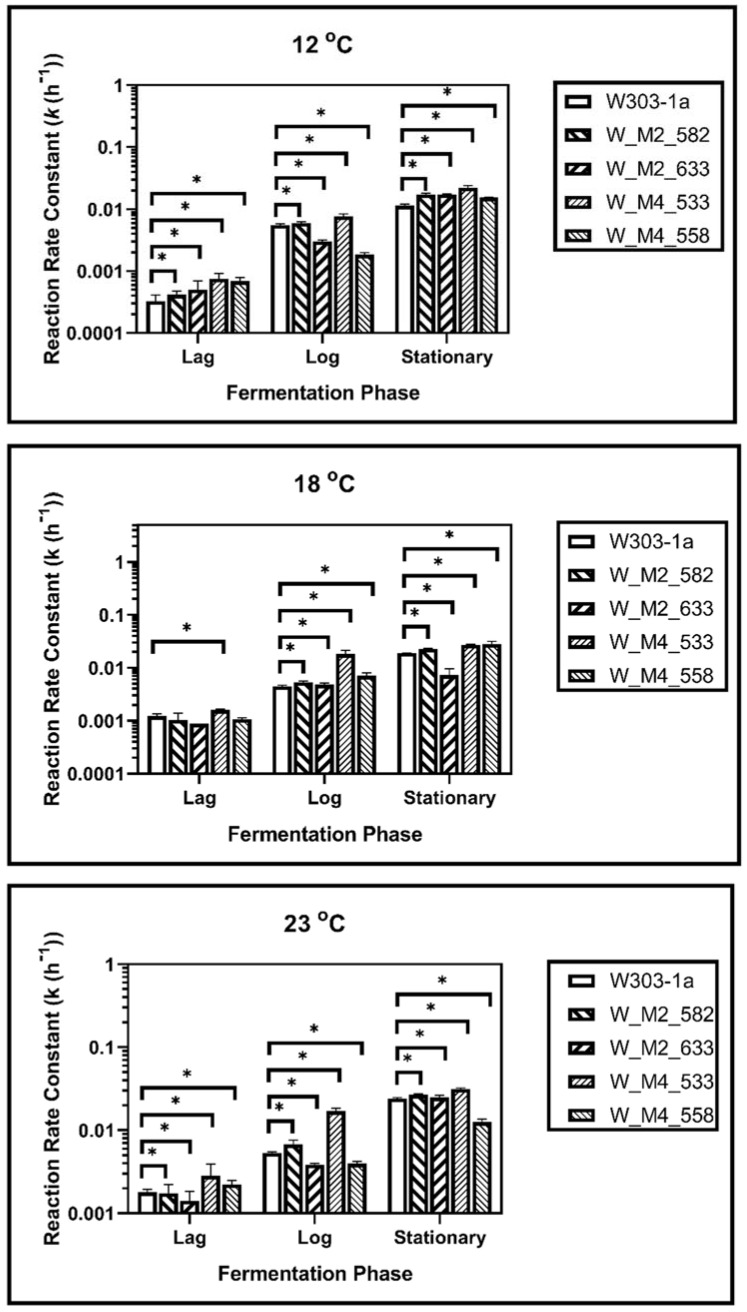
Reaction rate constants of hexose catabolism during alcoholic fermentation regarding phases for each strain at 12, 18 and 23 °C. The statistically significant differences are nominated by asterisks: (*: *p* < 0.05).

To verify this the sugar content during fermentation was analyzed using HPLC. The analysis also recognized the three phases of fermentation (lag phase, log phase and stationary phase). The reaction rate constants of total sugar catabolism for lag phase, log phase and stationery/death phase are *k*_1_, *k*_2_, and *k*_3_, respectively. The calculated reaction rate constants are presented in Table 3 as well as in Figure 7.

**Figure 8:**
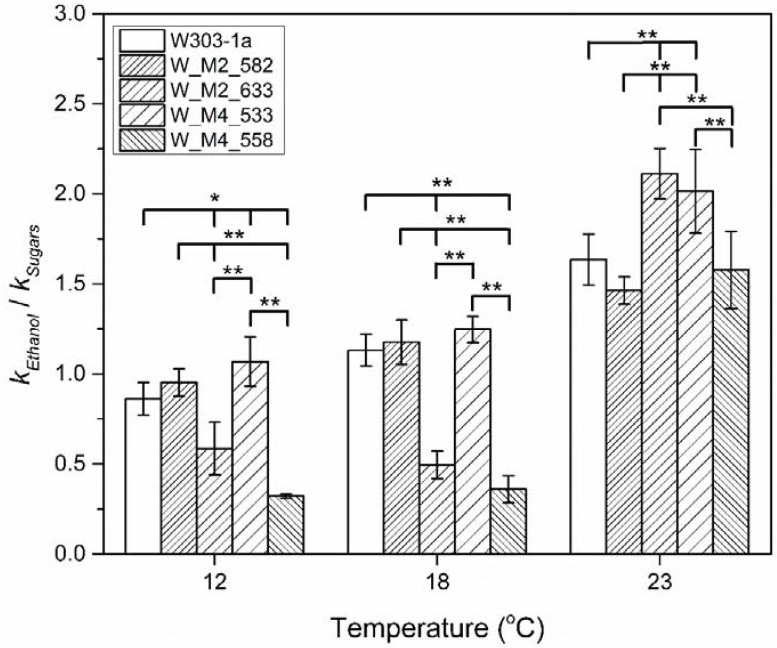
Reaction rate constants ratio, of ethanol formation vs. hexose catabolism during stationary/death phase for each strain at 12, 18 and 23 °C. The statistically significant differences are nominated by asterisks: (* *p* < 0.05, ** *p* < 0.01).

Comparing the results in Figure 4 and Figure 7, it can be observed that there are differences in the reaction rate constants, indicating probable different rate functions of the Embden–Meyerhof (glycolysis) pathway and its subsequent decarboxylation to acetaldehyde and reduction of the later to ethanol. The catabolism of one hexose molecule results in the formation of two ethanol molecules. Bearing this in mind and with the assumption that sugars are not used by the cell for other vital cell functions, the reaction rate constant of ethanol formation should have a value approximately double than of the sugars’ catabolism. As shown on Figure 8 the ratio of ethanol formation reaction rate constant vs. glucose catabolism reaction rate constant is lower than the theoretical value (approximately 2) either for wild type or mutant strains and especially at lower temperatures.

This indicates that cell regulates the reaction rate of hexoses to ethanol transformation according to its needs. Additionally, there is an indirect proof of the assumption that the mutations that are discussed in this paper, not only affect the reaction rate constant of ethanol production but also affect the cells’ ability to catabolize carbohydrates (Figure 8). This kind of analysis also confirms the abovementioned results whereby substitution of serine residue in Msn4 at position 533 increases the cell ethanol production, especially, at low temperatures, during stationary phase where the maximum ethanol formation is observed. Of importance are the results of W_M2_633 and W_M4_558 strains. These strains, especially the latter one, showed a significant decrease in reaction rate in producing ethanol compared to that of hexose that they catabolize, at 18 and 12 °C. This might imply that the cells choose to use the available hexose reserves according to their needs of survival.

Many fruits have a rich content in sugars and especially in glucose and fructose. Yeasts have the ability to ferment both of these hexoses, with the majority of them showing differential preference in glucose utilization and fermentation, which is associated with the genetic background of the cell [22,23,24]. The reaction rate constant of fructose and glucose fermentation was calculated for each strain (*k*_1F_, *k*_2F_, *k*_3F_, *k*_1G_, *k*_2G_ and *k*_3G_) (Table 4 and Table 5). These results, as expected, indicated that there are differences in glucose utilization of mutants compared to wild type, demonstrating that modifications on Msn2/Msn4 transcription factors alter the Embden–Meyerhof pathway function.

Additionally, it can be observed that, despite the elevated reaction rate constants for the utilization of both hexoses for all mutants, these differences are not of the same extent. For instance, at 18 °C, W_M4_533 seems to have almost the same ability to ferment glucose compered to W303-1a during lag phase, whilst it manifests a double reaction rate constant of fructose catabolism. This might imply a different cell function regarding the entrance of fructose into glycolysis.

**Figure 9:**
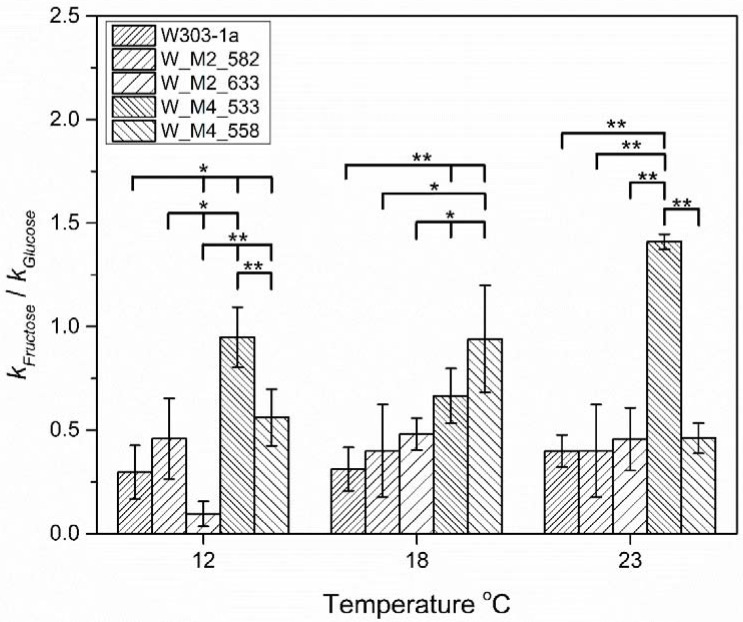
Reaction rate constants ratio, of fructose vs. glucose catabolism during lag phase for each strain at 12, 18 and 23 °C. The statistically significant differences are nominated by asterisks: (* *p* < 0.05, ** *p* < 0.01).

As the critical phase of hexose utilization during fermentation is the lag phase, where the cell adapts to the new environment and initiates fermentation, the ratios of reaction rate constant of fructose vs. reaction rate constant of glucose (*k*_1F_/*k*_1G_) were calculated for each strain and temperature (Figure 9). As can be observed, the substitution of S533A in Msn4 lead to the increase of the utilization of fructose during lag phase at all tested temperatures. At 18 °C, it possesses the second place behind W_M4_558 without showing significant difference from it, whereas at 12 and 23 °C, it is the better than any other.

Another interesting result is the significant inability of W_M2_633 to utilize fructose at the lowest tested temperature during lag phase. This inability is abolished as the temperature rises, implying that the hexose utilization is affected by genetic as well as environmental factors.

## 4. Discussion

Alcoholic fermentation is a complicated procedure, as the conversion of carbohydrates to ethanol requires the formation and simultaneous usage of many intermediate molecules. This process is regulated from an even more complicated protein network through several protein interaction and modifications. During alcoholic fermentation yeast cells are exposed to a stressful environment in which they have to adapt in order to survive and ferment. Msn2/4 transcription factors play a pivotal role in the yeast’s stress response mechanism, cell cycle regulation, survival and metabolism regulation [9].

Considering these facts, the scope of this work was the investigation of specific serine residues replacement of these two proteins, on having the potential to change the alcoholic fermentation efficiency of yeast. The substrate used for testing this hypothesis was reconstituted Corinthian currants must. The analysis of the product formation using a sophisticated analytical technique as RFGC, revealed that the substitution of serine residue to alanine at position 533 in Msn4 confers to the cell a significant increment of fermentation rate at low temperature (12 °C) as well as in medium temperature (18 °C). A similar effect but to a lesser extent was proven to occur when serine at position 582 in Msn2 was substituted by an alanine. These results were confirmed by analysis based on the Arrhenius model. The determination of activation energy was shown that S533A substitution result in the generation of a yeast strain which more efficiently adapts to low temperatures.

On the contrary, strains W_M2_633 and W_M4_558 were proven to increase the activation energy of alcoholic fermentation. All these results have given rise to a question about the ability of the strains to use and catabolize carbohydrates. Were the observed differences in activation energy of alcoholic fermentation, a result in different ability of each strain to catabolize glucose and/or fructose? In order to answer these questions, analyses of the glucose and fructose consumption were performed. Our results clearly show that S533A substitution in Msn4 confers the cell the ability to use the catabolized sugars more efficiently than wild type and other mutants, especially at 12 °C, enhancing its ability to adapt and to ferment more efficiently at low temperatures.

The usage of different hexoses during fermentation and especially during its initiation is of major scientific and industrial importance. It is known that *S. cerevisiae* in most of the cases prefers glucose over fructose. The consequence of this is the early depletion of glucose during fermentation and subsequent possibly high residual fructose levels. Since fructose is approximately twice as sweet as glucose this may result in undesirable sweetness in dry wines that have completed fermentation [23,25]. As Msn2/4 are transcription factors that regulate a plethora of genes, and their action is regulated by the presence of glucose the effect of the substitutions studied in this work is of major interest. The calculation of the ratio of the catabolic reaction rate constant of fructose vs. this of glucose confirms that alterations in Msn2/4 result in differences in the utilization of these two major fruit hexoses. Our results reveal that W_M4_533 strain displays an improved utilization of fructose. All these results clearly indicate that Msn2/4 transcription factors are of major importance regarding alcoholic fermentation, as different modifications will result in different phenotypes. This observation can help in the improvement of yeast strains that are used in the food industry.

## Figures and Tables

**Table 1:** Strains used in the current study.

Strain	Genotype	Ref.
W303-1a	*MAT* a, *ura3–1*, *leu2–3*, *his3–11*, *trp1–1*, *ade2–1*, *can1–1000*	[10]
W_M2_582	*MAT* a, *ura3–1*, *leu2–3*, *his3–11*, *trp1–1*, *ade2–1*, *can1–1000, msn2-S582A*	[10]
W_M2_633	*MAT* a, *ura3–1*, *leu2–3*, *his3–11*, *trp1–1*, *ade2–1*, *can1–1000, msn2-S633A*	[10]
W_M4_533	*MAT* a, *ura3–1*, *leu2–3*, *his3–11*, *trp1–1*, *ade2–1*, *can1–1000, msn4-S531A*	This Study
W_M4_558	*MAT* a, *ura3–1*, *leu2–3*, *his3–11*, *trp1–1*, *ade2–1*, *can1–1000, msn4-S558A*	[10]

**Table 2:** Primers used for the generation of MSN2 and MSN4 mutants.

*Primer*	*Sequence*
MSN4_URA3_F	5′CAGTTCGGCTTTTTTTTCTTTTCTTCTTATTAAAAACAATATAATGTCGAAAGCTACATATAAGG 3′
MSN4_URA3_R	5′CCGTAGCTTGTCTTGCTTTTATTTGCTTTTGACCTTATTTTTTTTAGTTTTGCTGGCCGCATC 3′
MSN4_533_F	5′ GAAGAAGAAAGTCGTCAGCTGTTACTTTAAGTCCAAC 3′
MSN4_533_R	5′ GTTGGACTTAAAGTAACAGCTGACGACTTTCTTCTTC 3′
MSN4_F	5′CAGTTCGGCTTTTTTTTCTTTTCTTCTTATTAAAAACAATATAATGCTAGTCTTCGGACCTAA 3′
MSN4_R	5′CCGTAGCTTGTCTTGCTTTTATTTGCTTTTGACCTTATTTTTTTCAAAAATCACCGTGCTTTTTGTG 3′

**Table 3:** Calculated hexose consumption reaction rate constants values (*k*) for each strain during fermentation phases at 12, 18 and 23 °C. The * indicates the observed statistically significant differences between genetically modified strains and wild type (*p* < 0.05).

		W303-1a		W_M2_582		W_M2_633		W_M4_533		W_M4_558	
Temp (°C)	Phase	*k* (h^−1^) × 10^−3^	SEM^*^	*k* (h^−1^) × 10^−3^	SEM	*k* (h^−1^) × 10^−3^	SEM	*k* (h^−1^) × 10^−3^	SEM	*k* (h^−1^) × 10^−3^	SEM
12	Lag	0.327	0.085	0.416 *	0.057	0.504 *	0.198	0.754 *	0.164	0.688 *	0.099
	Log	5.550	0.269	6.020 *	0.223	3.030 *	0.167	7.660 *	0.768	1.840 *	0.146
	Stationary	11.46	0.678	17.26 *	0.856	17.32 *	0.504	22.24 *	1.990	15.40 *	0.292
18	Lag	1.230	0.133	1.040	0.353	0.884	0.001	1.600 *	0.075	1.080	0.068
	Log	4.480	0.173	5.340 *	0.231	5.690 *	0.286	18.26 *	3.230	7.050 *	0.921
	Stationary	18.85	0.208	22.86 *	0.352	22.57 *	1.510	27.08 *	0.453	28.25 *	3.44
23	Lag	1.790	0.167	1.750 *	0.488	1.420 *	0.416	2.840 *	1.070	2.230 *	0.279
	Log	5.370	0.102	6.780 *	0.866	3.850 *	0.163	17.03 *	1.420	3.960 *	0.268
	Stationary	24.05	0.649	26.90 *	0.784	24.91 *	1.520	31.42 *	0.867	12.65 *	0.940

SEM: Standard error of the mean value.

**Table 4:** Calculated glucose consumption reaction rate constants values (*k*) for each strain during fermentation phases at 12, 18 and 23 °C. The * indicates the observed statistically significant differences between genetically modified strains and wild type (*p* < 0.05).

		W303-1a		W_M2_582		W_M2_633		W_M4_533		W_M4_558	
Temp (°C)	Phase	*k* (h^−1^) × 10^−3^	SEM	*k* (h^−1^) × 10^−3^	SEM	*k* (h^−1^) × 10^−3^	SEM	*k* (h^−1^) × 10^−3^	SEM	*k* (h^−1^) × 10^−3^	SEM
12	Lag	0.337	0.126	0.623 *	0.087	1.090 *	0.297	0.894 *	0.034	1.130 *	0.126
	Log	2.340	0.355	9.780 *	0.460	3.610 *	0.559	6.760 *	0.803	2.860 *	0.190
	Stationary	13.72	0.537	20.08 *	1.700	13.05	0.595	21.92 *	0.480	16.90 *	0.275
18	Lag	1.960	0.068	2.600 *	0.749	1.510 *	0.093	2.090	0.191	1.670	0.112
	Log	6.580	0.152	10.80 *	0.791	9.830	0.411	17.80 *	0.120	10.00 *	1.050
	Stationary	25.76	0.428	20.63 *	0.539	23.00 *	0.686	22.75 *	0.307	33.90 *	1.050
23	Lag	2.710	0.294	2.600	0.749	1.670 *	0.151	1.440 *	0.032	4.66 *	0.260
	Log	9.000	1.001	10.78 *	0.791	5.600 *	0.155	14.20 *	2.550	15.65 *	2.840
	Stationary	30.92	1.310	24.62 *	0.894	23.89 *	1.880	40.70 *	0.950	47.45 *	5.180

**Table 5:** Calculated fructose consumption reaction rate constants values (*k*) for each strain during fermentation phases at 12, 18 and 23 °C. The * indicates the observed statistically significant differences between genetically modified strains and wild type (*p* < 0.05).

		W303-1a		W_M2_582		W_M2_633		W_M4_533		W_M4_558	
Temp (°C)	Phase	*k* (h^−1^) × 10^−3^	SEM	*k* (h^−1^) × 10^−3^	SEM	*k* (h^−1^) × 10^−3^	SEM	*k* (h^−1^) × 10^−3^	SEM	*k* (h^−1^) × 10^−3^	SEM
12	Lag	0.100	0.006	0.285 *	0.082	0.102	0.038	0.848 *	0.098	0.634 *	0.084
	Log	2.790	0.174	2.960	0.121	1.470 *	0.197	4.960 *	0.588	4.150 *	0.282
	Stationary	13.22	0.947	17.07 *	0.789	15.35 *	1.250	22.45 *	2.200	15.29 *	0.312
18	Lag	0.610	0.185	1.040 *	0.281	0.725	0.071	1.390 *	0.149	1.570 *	0.327
	Log	3.380	0.120	4.440 *	1.090	3.530 *	0.426	7.420 *	0.004	8.340 *	0.934
	Stationary	17.34	0.352	24.58 *	1.650	21.38 *	0.130	26.91 *	0.355	28.10 *	3.600
23	Lag	1.08	0.089	1.040 *	0.281	0.763 *	0.184	2.030 *	0.006	2.150 *	0.220
	Log	3.520	0.216	4.440 *	1.090	4.010	0.404	11.59 *	1.350	12.59 *	1.320
	Stationary	20.08	1.620	24.58 *	1.650	24.49 *	1.660	31.17 *	0.413	38.86 *	2.580

## Data Availability

Data is contained within the article.
